# The anti-apoptotic and anti-inflammatory effect of *Lactobacillus acidophilus* on *Shigella sonnei* and *Vibrio cholerae* interaction with intestinal epithelial cells: A comparison between invasive and non-invasive bacteria

**DOI:** 10.1371/journal.pone.0196941

**Published:** 2018-06-06

**Authors:** Shabnam Zeighamy Alamdary, Bita Bakhshi, Sara Soudi

**Affiliations:** 1 Department of Bacteriology, Faculty of Medical Sciences, Tarbiat Modares University, Tehran, Iran; 2 Department of Immunology, Faculty of Medical Sciences, Tarbiat Modares University, Tehran, Iran; Virginia Polytechnic Institute and State University, UNITED STATES

## Abstract

The aim of this study was to compare the effect of *Lactobacillus acidophilus* on the attachment, invasion, and interaction of *Shigella sonnei* and *Vibrio cholerae* with Caco-2 epithelial cells. Also, the anti-apoptotic and anti-inflammatory effect of *L*. *acidophilus* was investigated on *S*. *sonnei* and *V*. *cholerae* interaction with Caco-2 cells as the representatives of invasive and non-invasive intestinal bacteria. It was found that pretreatment with *L*. *acidophilus* significantly prevented from adherence and internalization of *S*. *sonnei*/*V*. *cholerae* and reduced the expression of tumour necrosis factor-α and interleukin-8 in host cells. No significant difference was observed in inhibitory effect of Lactobacilli in *V*. *cholerae* and *S*. *sonnei* attachment, emphasizing on the role of lactobacilli as a physical barrier in inhibiting direct contact with host cell by competitive exclusion, which may affect attachment and subsequent internalization of both invasive and non-invasive pathogenic bacteria in a same scale. The evaluation of early and late apoptosis in Caco-2 cells exposed to *V*. *cholerae/S*. *sonnei* and pretreated by *L*. *acidophilus* indicated no remarkable difference in *L*. *acidophilus* anti-apoptotic effect on Caco-2 cells against invasive and non-invasive bacterial infection. Moreover, *L*. *acidophilus* by itself showed no apoptotic effect on Caco-2 cells. Statistical analysis revealed that *L*. *acidophilus* in *S*. *sonnei* infected cells was able to reduce pro-inflammatory immune responses (TNF-α, IL-8 and IL-1β) and NO and PGE_2_ secretion more strongly compared with *V*. *cholerae* infected cells. These data showed for the first time that the protective effect of Lactobacilli, as a probiotic bacterium, in interaction suppression was more in invasive bacteria including *S*. *sonnei* than in non-invasive *V*. *cholerae*.

## Introduction

Shigellosis is an acute infectious colitis caused by *shigella* spp. organisms. This diarrheal disease is a global human health problem in both developing and industrialized countries, and it is estimated that shigellosis causes over than one million deaths per year, most of which are patient children under 5 years old. *Shigella sonnei* are rod-shape, non-motile, non-flagellated, facultative anaerobic, Gram-negative, and lactose-fermenting bacteria that cause dysentery by invading the colonic mucosa from the basolateral surface; multiplying within colonic epithelial cells; causing cell death; spreading laterally; infecting and killing adjacent epithelial cells; causing mucosal ulceration, inflammation, and bleeding. These organisms are typically confined to the epithelial layer of the colonic mucosa [1–3].

With a different mode of action, *Vibrio cholera*e causes a fetal diarrheal disease which is manifested with acute watery diarrhea. *V*. *cholerae* is halophilic, highly motile, curved and Gram-negative rod. During the course of disease, *V*. *cholerae* is ingested and survives the low pH of the stomach to colonize the host small intestine. During colonization, *V*. *cholerae* uses motility and mucinase to penetrate the mucus layer of the intestine and gain access to the underlying epithelial cell layer. Indeed, *V*. *cholerae* as a classical agent of secretory diarrhea [4] and *S*. *sonnei* as an agent of inflammatory diarrhea produce choleratoxin and shigatoxin [3], respectively, by colonizing to epithelial surface, they are responsible for inflammatory destruction and simultaneously the extent of the elicited innate responses. Although the use of various antimicrobial agents is the first step to reduce illness duration and possibly the transmission of these pathogens, high rates of drug resistance have limited the choice of antimicrobial agents. Lactobacilli as non-spore-forming, Gram-positive, non-motile rods are recognized as natural components of the colonic microbiota and as probiotic and friendly bacteria. They have been tested in the prevention and treatment of gastrointestinal diseases [5, 6]. Since intestinal epithelial cells can respond to intestinal pathogens by producing an array of cytokines and chemokines which are associated with host immune responses [7], some strains of lactobacilli have been investigated for their cytoprotective effects on intestinal epithelial cells by regulating cytokine and chemokine production [5, 8, 9].

Indeed, this study allows for a better understanding of how the commensal Lactobacilli contribute to the homeostasis of the host intestinal tract. Indeed, the main goals of this research were (i) investigating the protective effect of *L*. *acidophilus* on viability of Caco-2 cells (human colon adenocarcinoma cells which are broadly known as a model of absorptive and defensive properties of the intestinal mucosa) infected by *V*. *cholerae* (as a non-invasive small intestine pathogen model) and *S*. *sonnei* (as an invasive colon pathogen model), (ii) enumerating the inhibitory role of *L*. *acidophilus* in *V*. *cholerae* adherence to Caco-2 cells in comparison with *S*. *sonnei*, (iii) comparison of the relative apoptosis levels induced in *L*. *acidophilus*-treated Caco-2 cells following *V*. *cholerae* and *S*. *sonnei* exposure, (iv) exploring the effect of *L*. *acidophilus* on pro-inflammatory markers expression (IL-8, TNF-α and IL-1β) and NO and PGE_2_ releases in *V*. *cholerae* infected Caco-2 cells in comparison with *S*. *sonnei*.

## Results

### Protective effect of *L*. *acidophilus* in Caco-2 cells infected with *S*. *sonnei/V*. *cholerae*

**Fig 1:**
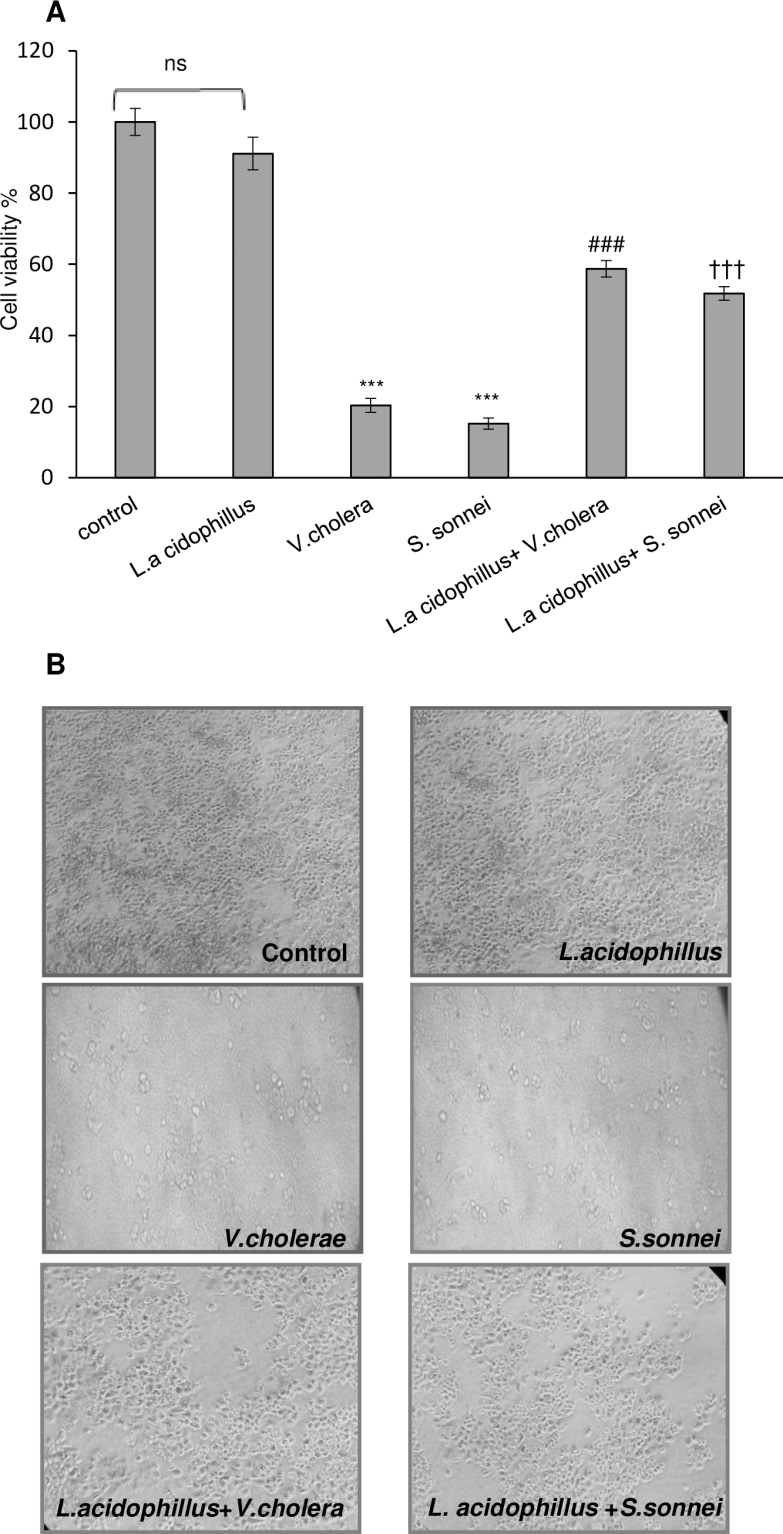
The effect of *V*. *cholerae*, *S*. *sonnei* and *L*. *acidophilus* on cell viability. (A) cell viability of Caco-2 cells infected by *V*. *colarae*/ *S*. *sonnei* was determined by MTT assay, Each value represents the mean ±SEM (n = 3). ***p < 0.001 compared with control (untreated Caco-2cells) by one way ANOVA with Tukey’s post hoc test. ^###^p < 0.001 significantly different of *L*.*a*+*V*.*c*+Caco-2 group from *V*.*c*+Caco-2 group. ^†††^p < 0.001 significantly different between *L*.*a*+*S*.*s*+Caco-2 group with *S*.*s*+Caco-2 (by unpaired two-tailed Student’s *t* test). All experiments were repeated three times. *L*.a: *Lactobacillus acidophilus*, *V*.*c*: *Vibrio cholerae* and *S*.*s*: *Shigella sonnei*, ns: none significant. (B) Morphological evaluation of Caco-2 cells under the above-mentioned treatment by light microscopic observation. *L*.a: *Lactobacillus acidophilus*, *V*.*c*: *Vibrio cholerae* and *S*.*s*: *Shigella sonnei*.

According to the experimental groups, Caco-2 cells were plated in 96-well plate and exposed to 10^7^ *L*. *acidophilus* for 2 hours before 4 h exposure to *S*. *sonnei* and *V*. *cholerae*. After 4 h, the cells were assayed for cell viability with MTT. Based on the results shown in (Fig 1A), pretreatment with *L*. *acidophilus* increased cell viability to 51.81 and 58.72% against *S*. *sonnei* and *V*. *cholerae* infections, respectively. Moreover, *L*. *acidophilus* alone did not have any cytotoxic effect on Caco-2 cells. Statistical significant increase (*p* < .001) in Caco-2 cells viability was observed in both test groups (*L*.*a+V*.*c* and *L*.*a+S*.*s*) compared to control group with no significant difference between the 2 test groups. The protective effect of *L*. *acidophilus* was further confirmed by morphological observation (Fig 1B).

### Effect of *L*. *acidophilus* pretreatment on attachment and internalization of *S*. *sonnei* /*V*. *cholerae* to Caco-2 cells

**Fig 2:**
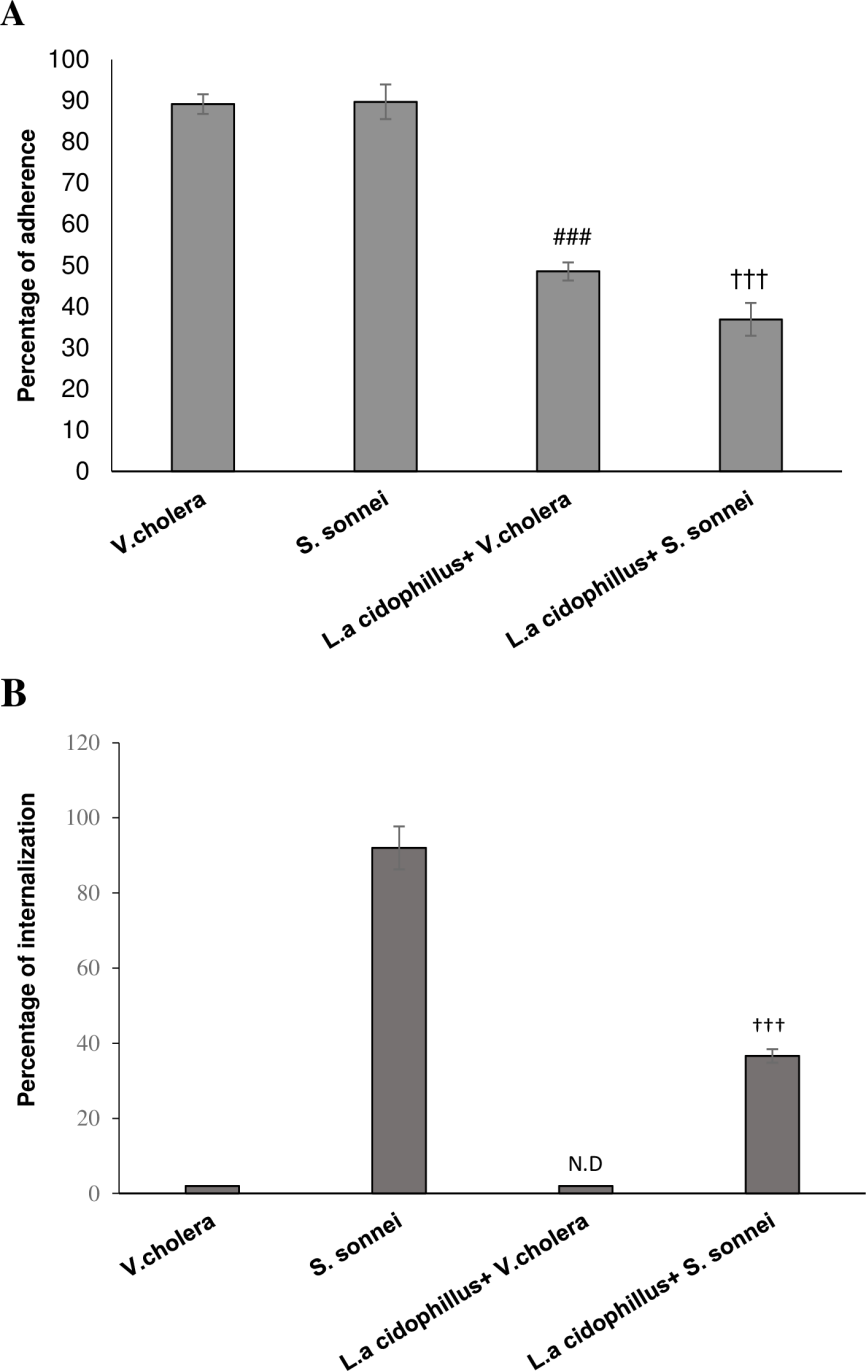
(**A and B) Effect of pretreatment of *L*. *acidophilus* on its ability to inhibit *V*. *colarae/ S*. *sonnei* adherence and invasion to Caco-2 cells.** ND means Not Detectable. Each value indicates the mean±SEM from three independent experiments and three replicates (n = 3). ^###^p < 0.001 significantly different of *L*.*a*+*V*.*c*+Caco-2 group from *V*.*c*+Caco-2 group. ^†††^p < 0.001 significant difference between *L*.*a*+*S*.*s*+Caco-2 group and *S*.*s*+Caco-2 (by unpaired two-tailed Student’s *t* test). *L*.*a*: *Lactobacillus acidophilus*, *V*.*c*: *Vibrio cholerae* and *S*.*s*: *Shigella sonnei*.

Pretreatment of Caco-2 cells with *L*. *acidophilus* significantly (*p* < .001) decreased the adherence of both *S*. *sonnei /V*. *cholerae* to Caco-2 cells compared to control group. In cells pretreated with *L*. *acidophilus*, adherence rate of *S*. *sonnei* was decreased from 89.72 to 36.91%. This decrease was from 89.17 to 48.54% for cells infected with *V*. *cholerae* after pretreatment step. (Fig 2A). No significant difference was observed between attachment reductions for the 2 pathogenic bacteria. Pretreatment of Caco-2 cells with *L*. *acidophilus* decreased significantly (*p* < .001) the internalization of *S*. *sonnei* from 92 (control) to 36% (test). No internalization was observed for *V*. *cholerae* in test or control group (Fig 2B).

### Assessment of apoptosis in *V*. *cholerae/ S*. *sonnei* infected Caco-2 cells pretreated with *L*. *acidophilus* by flow cytometry

**Fig 3:**
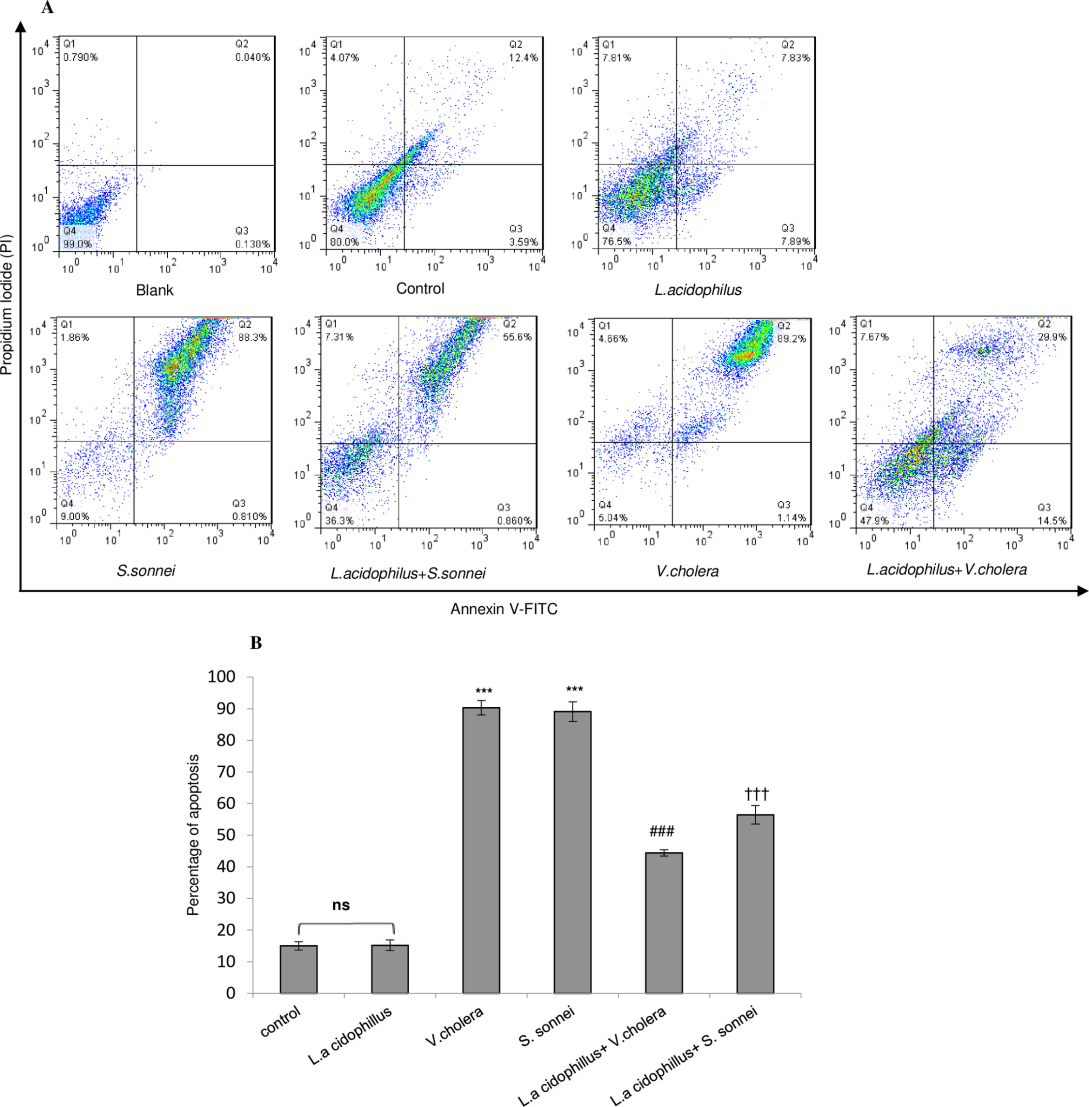
Annexin V-FITC/PI staining and flow cytometry assay to determine the proportion of apoptosis after pretreatment of Caco-2 cells with *L*. *acidophilus* and then infected by *V*. *colarae/ S*. *sonnei*. Images showed the typical morphologies of cells in the different regions. (A) Q1: Necrotic cells, Q2: Late apoptotic cells, Q2: Early apoptotic cells and Q4: Viable cells. *L*.*a*: *Lactobacillus acidophilus*, *V*.*c*: *Vibrio cholerae* and *S*.*s*: *Shigella sonnei*. (B)The pretreatment with *L*. *acidophilus* on Caco-2 cells exposed to *V*. *colarae/ S*. *sonnei* resulted in decreased apoptosis. Statistical analysis of representative images was performed with three independent experiments. The statistical significances were achieved when P <0.05 (***P <0.001). *** significantly different from control cells. ^###^ significantly different of *L*.*a*+*V*.*c*+Caco-2 from *V*.*c*+Caco-2 cells. ^†††^ significantly different of *L*.*a*+*S*.*s*+Caco-2 from *S*.*s*+Caco-2 cells *L*.*a*: *Lactobacillus acidophilus*, *V*.*c*: *Vibrio cholerae* and *S*.*s*: *Shigella sonnei*. ns: none significant.

The Caco-2 cells pre-treated with *L*. *acidophilus* (2 hours) before exposure to S. *sonnei /V*. *cholerae* showed lower apoptotic cells (56 and 43%, respectively) compared with un-treated S. *sonnei /V*. *cholerae* infected Caco-2 cells (89 and 90%, respectively) (*p* < .001) (Fig 3). No significant difference was observed in protectivity role of *L*. *acidophilus* against apoptosis of Caco-2 cells exposed to *V*. *cholerae* and *S*. *sonnei*.

### Real time-PCR for pro-inflammatory markers (IL-8, TNF-α and IL-1β)

**Fig 4:**
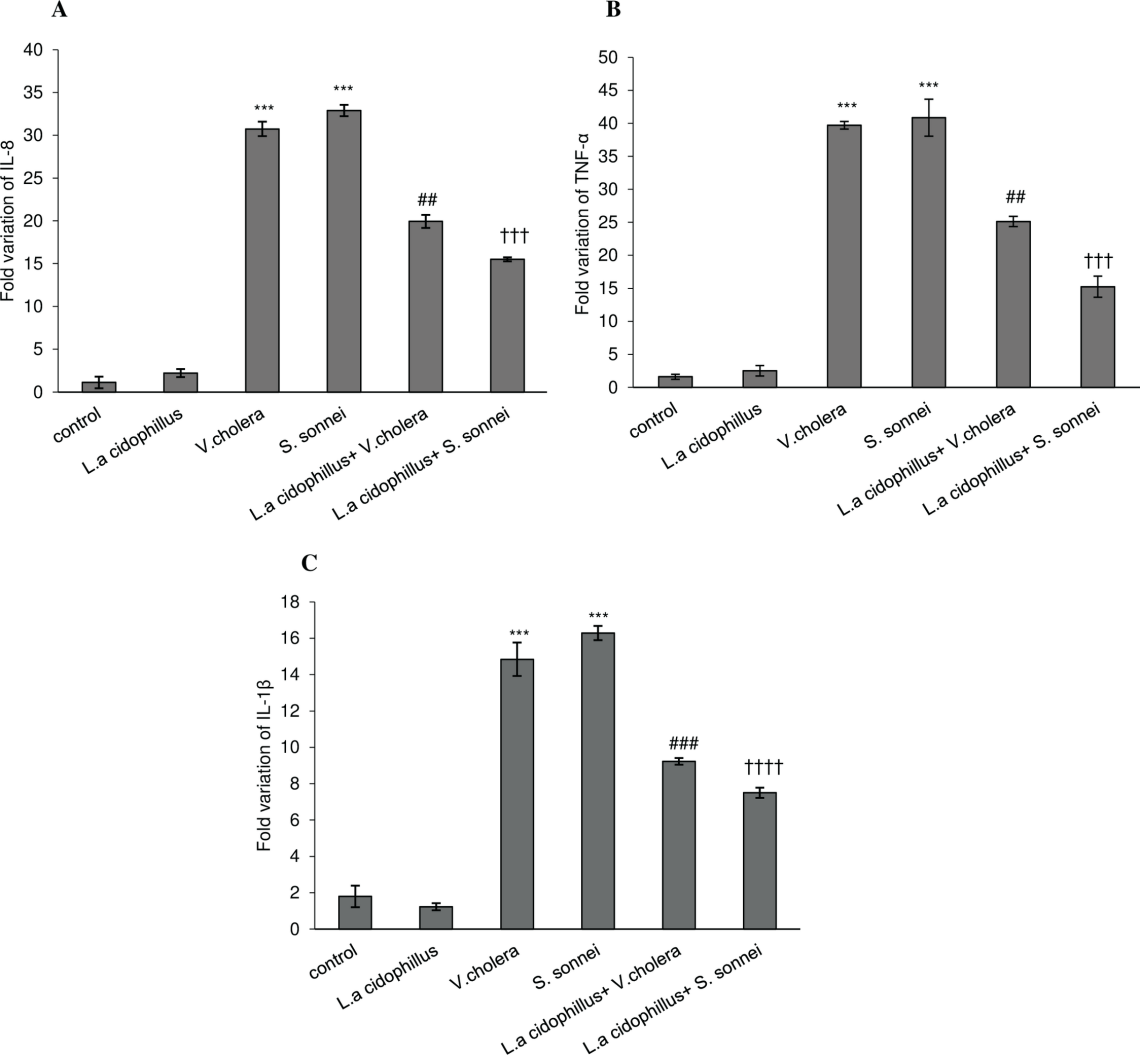
Expression of selected genes (IL-8. TNF-α and IL-1β) was determined by real-time quantitative polymerase chain reaction analysis in experimental cell groups. The fold-changes in expression of (A)IL-8, (B)TNF-α and (C)IL-1β after treatment by *L*. *acidophilus* was normalized to GAPDH and compared in infected and uninfected Caco-2 cells. The fold-changes in genes expression were represented from at least 3 independent experiments. The statistical results were remarkable when P <0.05. (***P <0.001) significantly different from control cells. ^##^ or ^###^ significant difference between *L*.*a*+*V*.*c*+Caco-2 and *V*.*c*+Caco-2 cells. ^†††^ or ^††††^ significant difference between *L*.*a*+*S*.*s*+Caco-2 and *S*.*s*+Caco-2 cells *L*.*a*: *Lactobacillus acidophilus*, *V*.*c*: *Vibrio cholerae* and *S*.*s*: *Shigella sonnei*.

In *L*. *acidophilus* pretreated Caco-2 cells followed by S. *sonnei /V*. *cholerae* infection, the expression of IL-8, TNF- α, and IL-1β markers was down regulated compared to un-treated S. *sonnei /V*. *cholerae* infected Caco-2 cells (*p* < .001) (Fig 4A, 4B and 4C). As shown in (Fig 4A), in test group (L.a+V.c/L.a+S.s), IL-8 level was decreased by 2.3 and 1.5 fold, respectively, compared to untreated S. *sonnei /V*. *cholerae* infected controls (V.c/S.s). Moreover, TNF-α expression in Caco-2 cells was decreased by 2.7 and 1.5 fold, respectively, when cells were treated with *L*. *acidophilus* in comparison to *S*. *sonnei /V*. *cholerae* infected Caco-2 cells (Fig 4B). In (Fig 4C), IL-1β level was decreased by 2.2 and 1.6 fold, respectively, compared to controls (V.c/S.s) (S1 Table). The inhibitory effect of *L*. *acidophilus* on pro-inflammatory markers expression in *S*. *sonnei* infected Caco-2 cells was more significant than in *V*. *cholerae* infected Caco-2 cells (†*p* < .05).

### The effect of *L*. *acidophilus* on nitric oxide (NO) release in Caco-2 cells

**Fig 5:**
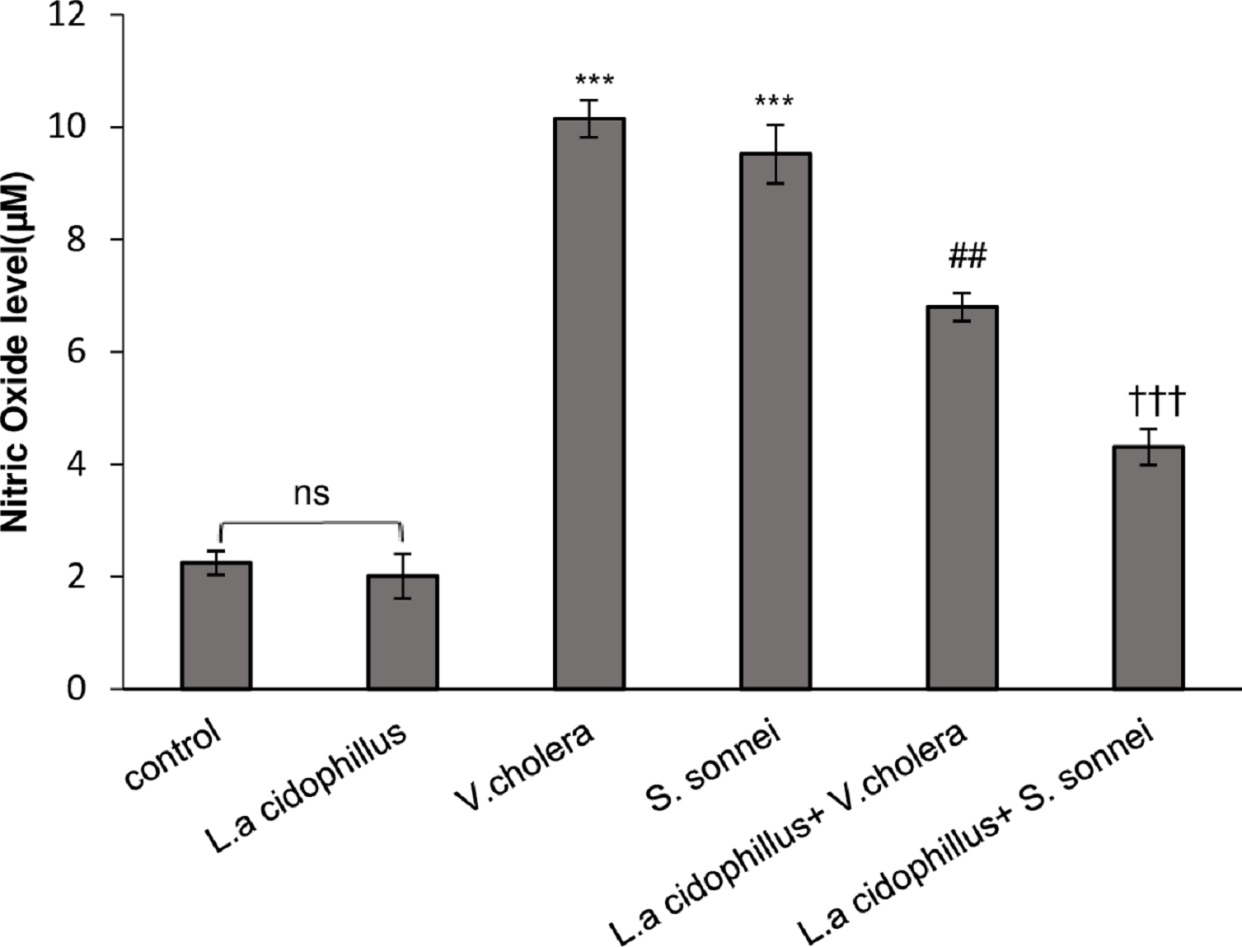
NO level in Caco-2 cells after *L*. *acidophilus* pretreatment and followed by *V*. *cholerae/ S*. *sonnei*. NO production after 24h, significantly decreased in comparison with *V*.*cholerae/ S*. *sonnei* infected cells alone. The mean of three independent experiments is shown. The significant differences were notabled when P <0.05. (***P <0.001) significantly different from control cells. ^##^significantly different of *L*.*a*+*V*.*c*+Caco-2 from *V*.*c*+Caco-2 cells. ^†††^ significantly different of *L*.*a*+*S*.*s*+Caco-2 from *S*.*s*+Caco-2 cells *L*.*a*: *Lactobacillus acidophilus*, *V*.*c*: *Vibrio cholerae* and *S*.*s*: *Shigella sonnei*. ns: none significant.

The level of NO production in supernatants of Caco-2 cells was increased 10.15 and 9.52 μM in response to *V*. *cholerae* and *S*. *sonnei* infection compared to un-infected Caco-2 cells (Fig 5). Pre-treatment of Caco-2 cells with *L*. *acidophilus* decreased the level of NO production 6.8 and 4.3 μM in response to *V*. *cholerae* and *S*. *sonnei* infections, respectively. Nitric oxide secretion in *S*. *sonnei* infected Caco-2 cells pretreated by *L*. *acidophilus* decreased 1.4 fold while this level was 2.2 fold in *V*. *cholerae* infected Caco-2 cells (Fig 5). The decreasing role of *L*. *acidophilus* on NO expression in *S*. *sonnei* infected Caco-2 cells was more significant than in *V*. *cholerae* infected Caco-2 cells (†*p* < .05).

### Caco-2 epithelial cell lines and PGE_2_ release in response to *V*. *cholerae* and *S*. *sonnei* infection

**Fig 6:**
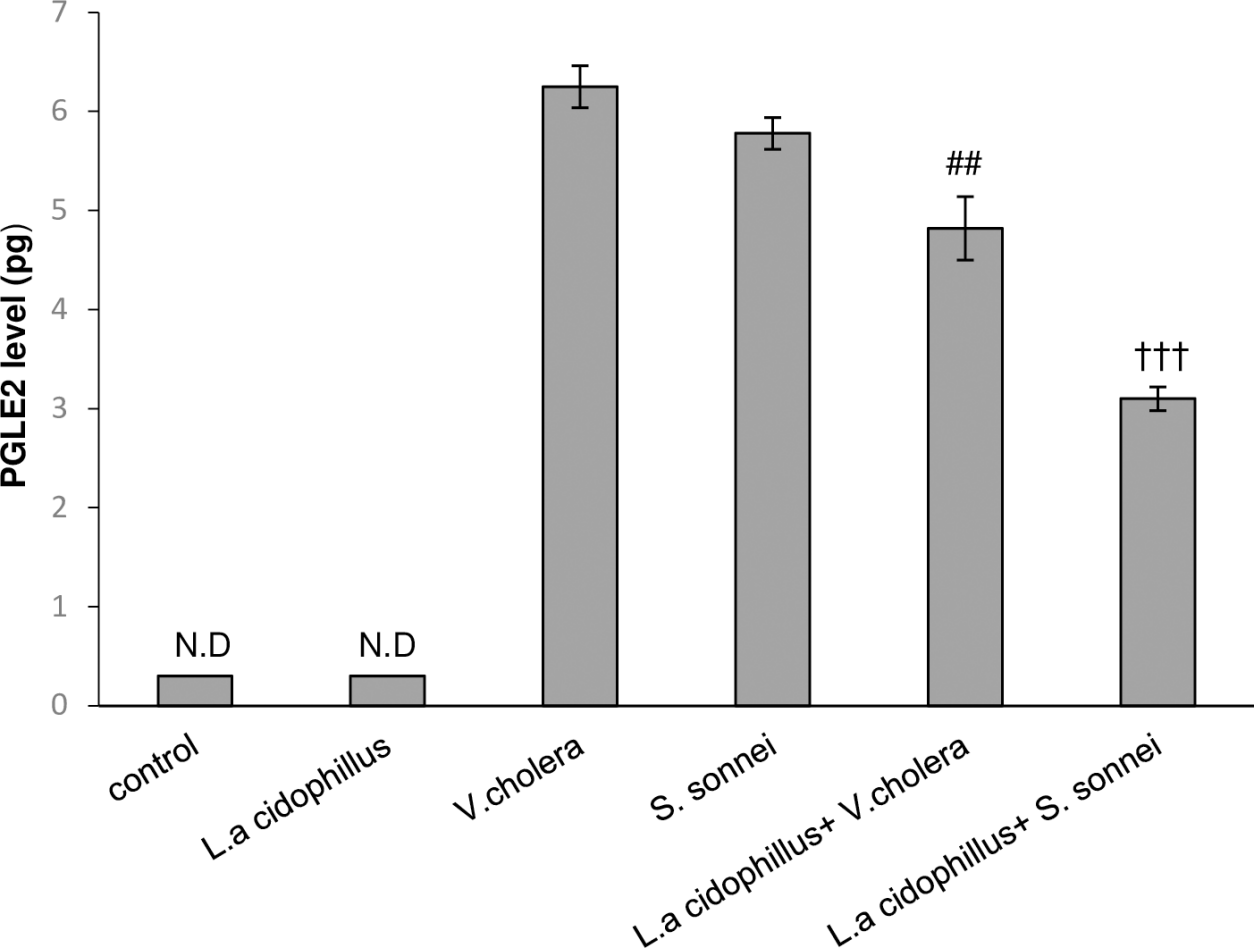
Effect of treatment with *L*. *acidophilus* on PGE2 secretion in Caco-2 epithelial cells. PGE2 secretion was significantly elevated following infection with *V*. *cholerae/ S*. *sonnei* and this effect was abolished when the cells were pretreated by *L*. *acidophilus*. The data are presented as mean ±SEM. The statistical data (by unpaired two-tailed Student’s *t* test) were signed when P <0.05. ^##^significantly different of *L*.*a*+*V*.*c*+Caco-2 from *V*.*c*+Caco-2 cells. ^†††^ significantly different of *L*.*a*+*S*.*s*+Caco-2 from *S*.*s*+Caco-2 cells *L*.*a*: *Lactobacillus acidophilus*, *V*.*c*: *Vibrio cholerae* and *S*.*s*: *Shigella sonnei*. ND: None Detectable.

The level of PGE_2_ secretion in infected Caco-2 cells with/without pretreatment by *L*. *acidophilus* are shown in (Fig 6). Caco-2 cells produced high level of PGE_2_ in response to *V*. *cholerae*/*S*. *sonnei* infection (6.25 and 5.78 pg, respectively), which was decreased to 4.8 and 3.1 pg in response to *L*. *acidophilus* pretreatment. Statistical analysis showed that inhibitory effect of *L*. *acidiphilus* on PGE_2_ production in *S*. *sonnei* infected Caco-2 cells was more significant than in *V*. *cholerae* infected Caco-2 cells (†*p* < .05).

## Discussion

Lactobacilli are normal commensal flora of the gut and as beneficial microbes are used in the therapy of gastrointestinal diseases and enhancement of intestinal health [10]. In the current study, we have investigated the protective effect of *L*. *acidophilus* in inhibition of interaction of *S*. *sonnei* as a colon-invasive pathogen and *V*. *cholerae* as a non-invasive pathogen of small intestine with Caco-2 intestinal epithelial cells.

Our results indicated that pretreatment of Caco-2 cells with *L*. *acidophilus* followed by *V*. *cholerae/S*. *sonnei* infection was resulted in a significant increase in viability of Caco-2 intestinal epithelial cells (2.9 and 3.3 folds, respectively). These data suggest that *L*. *acidophilus* has a strong protective effect on Caco-2 intestinal epithelial cells against *V*. *cholerae/S*. *sonnei* induced cell death with no significant difference between invasive and non-invasive bacteria under study.

The adherence and invasion of *V*. *cholerae/S*. *sonnei* to Caco-2 cells was reduced following pretreatment by *L*. *acidophilus*. Previous researchers established that *L*. *acidophilus* had the ability to bind to brush borders of human epithelial cells [11], in which calcium cations act as an ion bridge to connect the surface of epithelial cells and *L*. *acidophilus*[12]. Also, adherence of lactobacilli to the intestinal epithelial cells is considered to be essential for the exertion of the protective effect against enteropathogenic bacteria[13, 14]. Moorthy et al (2010) has indicated that treatment of epithelial cells by lactobacilli would reduce adherence and invasion of *S*. *dysenteriae* to epithelial cells, but the interaction remained to be understood and compared for *S*. *sonnei* and *V*. *cholerae* regarding their increasing occurrence in developed and developing countries[15] and considering their different mode of action and consequences. No significant difference was observed in inhibitory role of Lactobacilli in *V*. *cholerae* and *S*. *sonnei* attachment. The probable explanation is i) the role of lactobacilli as a physical barrier to inhibit direct contact with host cell by competitive exclusion, which may affect attachment and subsequent internalization of both colon-invasive and non-invasive pathogens of small intestine in a same scale, ii) bacteriocin production by Lactobacilli may have direct inhibitory effect on adherence of both invasive and non-invasive pathogenic bacteria to epithelial cells [16, 17], and iii) immunomodulation of cellular mechanisms by *Lactobacillus* probably interferes with pathogen adhesion to epithelium probably through induction of primary apoptosis [18, 19]; however, late apoptosis which is supposed to be induced by pathogenic bacteria is inhibited by anti-inflammatory role of Lactobacilli, applied by suppression of TNF-α production.

Although many studies have dealt with the role of lactobacilli, as a part of the intestinal microflora, in modulation of host immunological responses, *L*. *acidophilus* has showed dual manner in apoptosis proceeding of epithelial cells. The study by Shumei Wang (2014) showed that cell walls and cytoplasm extracted from Lactobacilli induced DNA damage and apoptosis in HT-29 cells [20]. Russo et al in 2007 showed that *L*. *rhamnosus* strain *GG* homogenate showed apoptotic induction action in human gastric cancer cells [21]. In addition, a research by Chandra et al in 2008 showed supernatant of *Lactobacillus reuteri* regulated cell proliferation in myeloid leukemia-derived cells by promoting apoptosis and enhancing pro-apoptotic MAPK signaling [22]. Moreover, Soltan Dallal et al. (2015) indicated that treatment of Caco-2 cell line with either *L*. *acidophilus* or *L*. *Casei* supernatants and extracts was able to increase apoptosis, and consequently, to hinder the cancer cell migrations and invasions [23]. Controversially, some other studies revealed that *L*. *acidophillus* S-layer proteins in Caco-2 cells infected by *Salmonella* protected cells against apoptosis [24]. In the present study, the process of early and late apoptosis was evaluated in Caco-2 cells exposed to *V*. *cholerae/S*. *sonnei* and pretreated by *L*. *acidophilus*. The obtained data indicated that *L*. *acidophilus* pretreatment caused 2.1 and 1.5 fold reduction in apoptosis rate of *V*. *cholerae* and *S*. *sonnei* infected Caco-2 cells, respectively. Moreover, the results revealed that *L*. *acidophilus* by itself had no apoptotic effect on Caco-2 cells. Furthermore, statistical comparisons showed no remarkable difference in *L*. *acidophilus* anti-apoptotic effect on Caco-2 cells between invasive and non-invasive bacterial infection.

One of the toxic chemical species elaborated by the host epithelial cells during bacterial infection is nitric oxide (NO) which acts as an early signal to activate an acute mucosal inflammatory response and enhance the ability of epithelial cells to produce cytokines that regulate mucosal immune responses [25, 26]. In parallel, it is stated that Lactobacilli could reduce nitric oxide release [27]. Upon stimulation by proinflammatory cytokines such as IL-1β and TNF-α, as an important lipit mediator, PGE2 synthesis is upregulated following Gram-negative and Gram-positive bacterial infection [28–31]. Statistical comparisons between *V*. *cholerae* and *S*. *sonnei* infected Caco-2 cells pretreated by *L*. *acidophilus* surprisingly established significant difference in reduction of proinflammatory markers (TNF-α, IL-8 and IL-1β) expression and NO and PGE_2_ secretion (*P*-value < .05). Considering the critical role of TNF-α in modulating host immune responses, subsequent decrease in cell apoptotic responses can be attributed to inhibitory role of Lactobacilli in TNF-α production. Apoptosis was decreased but not missed, suggesting that the remaining apoptotic cells themselves reduce proinflammatory cytokines production which was considered to be an active process mediated by the production of PGE2 and NO [32, 33]; However, the role of remaining apoptotic cells in reducing TNF-α production should not be ignored[34–36].

In conclusion, statistical analysis revealed that *L*. *acidophilus* in *S*. *sonnei* infected cells could reduce pro-inflammatory immune responses more strongly compared with *V*. *cholerae* infected cells. These data show for the first time the more protective effect of Lactobacilli, as a beneficial bacterium, in suppression of colon-invasive bacteria including *S*. *sonnei* compared with *V*. *cholerae*, the non-invasive pathogen of small intestine. It can be suggested that i) the invasive nature of *S*. *sonnei* is the notable point for explaining the more efficient contribution of *L*. *acidophilus* in protection of intestinal epithelial cells, ii) attachment and invasion is a more critical step affecting subsequent inflammatory responses in *S*. *sonnei* infection than in *V*. *cholerae*, or iii) the anti-inflammatory properties of Lactobacilli may affect the apoptotic response of epithelial cells probably through the reduction of TNF-α.

## Materials and methods

### Bacterial strains and growth conditions

This study was approved by Medical Ethics Committee of Tarbiat Modares University (Code: IR.MODARES.REC) before the study began. *S*. *sonnei* ATCC9290, kindly provided by WHO, *Lactobacillus acidophilus* ATCC314, obtained from Pasteur Institute of Iran as a gift, and *Vibrio cholerae* ATCC14035, obtained from archive of our laboratory, were used in this study. *S*. *sonnei* and *V*. *cholerae* were routinely grown on Brain Heart Infusion (BHI) Broth at 37°C for 18–20 h before use. *L*. *acidophilus* was grown on Man–Rogosa–Sharpe (MRS) agar at 37°C under microaerophilic condition with 5% CO_2_ for 18–20 h before use.

### Cell culture condition

Caco-2 cells purchased from Pasteur Institute of Iran were cultured in Dulbecco’s modified Eagle’s medium (DMEM) (Sigma, Aldrich) supplemented with 5% fetal bovine serum and 1% antibiotic mixture of penicillin-streptomycin and incubated in a humidified atmosphere at 37°C with 5% CO2. Towards confluency, Caco-2 cells started to polarize, acquiring a characteristic apical brush border with microvilli. Tight junctions were formed between adjacent cells, and macromolecules were sorted and maintained between apical and basolateral surfaces of confluent cells. However, markers of colonocytes are also present in Caco-2 cells [37].

Culture of intestinal epithelial cells on 3D chambers (Transwell) is similar to in vivo environments, and cells are better polarized with sorted distribution of certain molecules exposed, at the apical surface and basolateral surface only. But, intestinal epithelial cells in ordinary tissue culture (2D medium culture) have surface molecules distributed all around the cell. Since cells in monolayer culture with full confluency can form polarized and tight monolayers with sorting and maintaining of cell surface molecules, we explored cells in full confluency (90% confluent) for all trials.

### Design of cell groups for treatment

Intact untreated Caco-2 cells were served as control group (Group 1), cells incubated with *L*. *acidophilus* as Group 2, cells infected with *V*. *cholerae* as Group 3, cells incubated for 2 hours with *L*. *acidophilus* followed by the addition of *V*. *cholerae* as Group 4, cells infected with *S*. *sonnei* as Group 5, and cells pretreated for 2 hours with *L*. *acidophilus* followed by the infection with *S*. *sonnei* as Group 6.

### Measurement of cell viability

Cell viability was evaluated by the conventional MTT (3-[4, 5-dimethylthiazol-2-yl]-2, 5-diphenyl tetrazolium bromide) reduction assay. This was tested to assess the viability of Caco-2 cells after the infection time. Approximately, 2 × 10^4^ of Caco-2 cells per well were seeded into a 96-well plate for MTT assay. Cells were exposed to each of 3 bacterial species, separately, at varying MOIs of 1, 10, 20, 30, 40, 100 at 24, 48, and 72h exposure times. The dark blue formazan crystals formed in cells were dissolved using dimethyl sulfoxide (DMSO), and the absorbance was measured at 570 nm with an ELISA reader after 30 min. The cellular viability was calculated as the percentage of survival relative to the control cells, showing the best results at the MOI of 10, where 80–90% of the cells were found to be infected, with a cell viability of >80% at the 24h time point.

### Enumeration of *L*. *acidophilus*, *S*. *sonnei* and *V*. *cholerae* for attachment and invasion experiments, apoptosis and cytokine assays

One colony of Lactobacillus culture on MRS agar was added to 5 mL MRS broth and incubated in 37°C under microaerophilic condition for overnight. Approximately, 300μL of overnight culture of Lactobacillus on MRS broth was added to 5mL fresh MRS broth and incubated at 37°C. After 2, 3, 4, 6 and 8 h incubation, the optical density (OD) was measured at 625nm wavelength, and 10μL of culture was simultaneously serially diluted and cultured on MRS agar and incubated in 37°C for overnight. The plates with 30–300 colonies per plate were counted and the Colony forming unit (CFU) was calculated as: number of colony × 100× inverse dilution factor. A growth curve was plotted for CFU based on OD_625_. The same procedure was applied for calculating the CFU of *S*. *sonnei /V*. *cholerae* on BHI agar plate under aerobic condition, as well.

The attachment and invasion experiments and apoptosis and cytokine assays were performed in 6-well plates and seeded with approximately 7 × 10^5^ of Caco-2 cells per well to reach the confluency of 90%. All test groups were inoculated with 1×10^7^ CFU.mL^-1^ of *L*. *acidophilus* 2 hours before addition of 1×10^7^ CFU.mL^-1^ *S*. *sonnei* or *V*. *cholerae*. The 1×10^7^ CFU.mL^-1^ of each bacterial species was related to OD 0.8 and fulfilled the MOI 10. Accordingly, 1 × 10^7^ CFU *L*. *acidophillus*, *S*. *sonnei* or *V*. *cholerae* per mL (the OD_625_ of 1 ×10^7^ bacteria/mL was 0.8, approximately) was centrifuged at 3500 × g for 5 min. Each supernatant was poured off and the pellet was resuspended in DMEM (without penicillin and streptomycin) after two washing steps with DMEM solution. All experiments were repeated three times and the average value was recorded.

### Enumeration of *S*. *sonnei* and *V*. *cholerae* invasion to Caco-2 cells

The confluent Caco-2 cells in 6 well plates were prepared in 2 parallel sets, each set consisted of the six designed groups to enumerate the adherent and internalized *S*. *sonnei* and *V*. *cholerae*. After incubation of cells with 10^7^ CFU of *L*. *acidophilus* for 2 hours (MOI = 10), followed by infection with 10^7^ CFU of *S*. *sonnei* or 10^7^ CFU of *V*. *cholerae* for 5 h, one set of groups of 6-well plates were washed twice with DMEM medium and treated with gentamicin (250 μg.mL^-1^) for 1 h to kill the extra cellular bacteria. Subsequently, the wells were washed by PBS for three times and lysed with 0.5% Triton-X 100 in PBS. Cell lysates were harvested and cultured onto BHI agar after serial dilution and incubated at 37°C for 18–24 h to assess the number of internalized *S*. *sonnei* or *V*. *cholerae* strains. The percentage of internalization was calculated based on following formula: total count of internalized pathogenic bacteria/ total number of inoculated pathogenic bacteria (10^7^ CFU)×100.

### Enumeration of adherent *S*. *sonnei* and *V*. *cholerae* to Caco-2 cells

The second set of 6-well plates were washed by PBS for three times to remove un-adhered bacteria. The cells were lysed with 0.5% Triton-X 100 in PBS and the lysed cells were plated onto BHI agar and incubated at 37°C for 18–24 h to enumerate total of adherent plus internalized bacteria.

Number of internalized *S*. *sonnei* or *V*. *cholerae* was subtracted from the total number of intracellular plus adherent *S*. *sonnei* or *V*. *cholerae* to assess the adherent pathogenic bacteria. The percentage of adherence was calculated by formula: total count of adherent pathogenic bacteria/ total number of inoculated pathogenic bacteria (10^7^ CFU)×100.

### Flow cytometry-based quantification of apoptosis in Caco-2 cells by annexin V/propidium iodide staining

Annexin V is a member of the annexin family of intracellular proteins that binds to phosphatidyl serine (PS) in a calcium-dependent manner. In viable and healthy cells, PS is located in the inner membrane leaflet, but during early apoptosis, it is translocated to the external leaflet and becomes available for annexin V binding. Annexin V binding alone cannot differentiate between apoptotic and necrotic cells. The addition of PI enabled viable, early apoptotic, late apoptotic, and necrotic cells to be distinguished. All the operations were performed according to the manufacturer’s instructions (FITC Annexin V Apoptosis Detection kit, BioLegend, USA). Briefly, the cells in 6 designed groups were rinsed with PBS and resuspended in a binding buffer [4]. Then 5 mL of Annexin V (20 mg.mL^-1^) and 10 mL of propidium iodide (PI, 50 mg.mL^-1^) were added to the suspension and incubated for 15 min at room temperature and analyzed by flow cytometer.

### Total RNA isolation and cDNA synthesis

Confluent Caco-2 cells were incubated at 37°C for the infection period (6 designed groups). At the end of the infection, medium was removed and cells were washed by PBS for 3 times. The cell monolayers were lysed with Trizol reagent (Sigma Aldrich), after which chloroform was added. The resulting mixture was centrifuged, and the aqueous phase was added to a fresh tube to which 2-propanol was added and centrifuged. Total RNA was washed twice with 70% ethanol, dried, and solubilized in Diethyl pyrocarbonate (DEPC treated water). RNA was quantified by absorbance at OD260/280 and only samples with a ratio of 1.8–2.0 were used for cDNA synthesis.

Easy™ cDNA Synthesis Kit (ParsTous, IRAN) was used for synthesis of cDNA. According to protocol manual, template RNA, Random hexamer, DEPC treated water, and RT Premix (2X) were mixed and incubated for 10 minutes at 25°C, 1 hour at 50°C, and five minutes at 65°C. The resulting cDNA samples were stored at -20°C until required for later investigation.

### Real-time RT-PCR analysis

The real-time RT-PCRs were performed by Rotor-Gene Q (Qiagen, Germany). Each PCR reaction contained 10μL 5x Real-time PCR Master Mix (Ampliqon, Denmark), 2 μL cDNA template, 1 μL of each primer and 6 μL distilled water in a total reaction volume of 20 μL. PCR reactions were performed according to annealing temperature of each primer pairs (Table 1). Conventional SYBR green based real-time PCR was used for gene quantification. Equal amounts of RNA were converted into cDNA in each sample and pipetting errors was avoided. GAPDH mRNA expression was quantified as the normalizer in each sample and the ΔCt of each sample was calculated (CT _target_−CT _reference)_.

**Table 1:** Oligonucleotide primers used for Real-time PCR.

Gene product	Primer Sequence	Amplicon size
**IL-8**	Forward: 5′AAACCACCGGAAGGAACCAT3′	117 bp
	Reverse: 5′GCCAGCTTGGAAGTCATGT3′	
**TNF-α**	Forward: 5′CAGAGGGAAGAGTTCCCCAG3′	653 bp
	Reverse: 5′CCTTGGTCTGGTAGGAGACG3′	
**IL-1β**	Forward: 5′GCACGATGCACCTGTACGAT3′	70 bp
	Reverse: 5′AGACATCACCAAGCTTTTTTGCT3′	
**GAPDH**	Forward: 5′ GATCATCAGCAATGCCTCC3′	420 bp
	Reverse: 5′ TCCACGATACCAAAGTTGTC3′	

GAPDH is one of the most commonly used reference genes and a great majority of the most important scientific journals concerns its use through what is often referred as “classical” [38]. We used un-treated control (intact caco-2) culture, as the calibrator and ΔΔCt method was used to calculate the difference between treated and controls. Finally, the gene expression data were presented as the fold change relative to untreated control group after normalization with an endogenous reference gene (GAPDH). The manuscript was edited as suggested. Each real-time PCR reaction was performed in triplicate.

### Nitric oxide assay

Nitric oxide (NO) is a diatomic free radical that is extremely short lived in biological systems and produced by various epithelial cells in response to the inflammatory functions. NO concentration in the supernatant was determined by a method based on the Griess reagent (Sigma Aldrich). The Griess reagent is made up of a 1% solution of sulfanilamide in 5% phosphoric acid and 0.1% naphthyl ethylenediamine dihydrochloride in distilled water. The supernatants of experimental cell groups (6 designed groups) were collected after infection period. The NO was assayed by mixing the equal volumes of supernatants and the Griess reagent. The absorption at 540 nm was read by an ELISA reader, and calibration curve was generated by different concentrations of sodium nitrite.

### Prostaglandin E2 (PGE2) immunoassay

PGE2 concentration was assessed by immunoassay using the PGE2 ELISA Kit (Invitrogen, USA). According to the manufacturer instructions, a serial dilution was prepared from standards, and the supernatants from 6 designed groups were added into the appropriate wells, and blue PGE2-AP conjugate and yellow PGE2 antibody were added into each well and incubated at room temperature. Substrate solution was added to every well and incubated after the wells were rinsed with wash buffer for 2 times. Finally, stop solution was added to each well, and the absorbance of reaction mixture was detected at 405 nm.

### Data analysis

Data were presented as the mean±standard error of the mean (S.E.M.). Statistical significance was assessed using GraphPad Prism 5 (GraphPad Software, San Diego, CA, www.graphpad.com). One-way analysis of variance (ANOVA) with Tukey’s post hoc test were performed for multiple comparisons, Statistical significance for *L*. *acidophilus* imaging data was assessed by unpaired two-tailed Student’s *t* test. The statistical significances were achieved when *P* < .05 (*or # or †*P* < .05, ** or ## or ††*P* < .01, *** or ### or †††*P* < .001 and **** or #### or ††††*P* < .0001).

## Supporting information

S1 Table Quantitative expression of IL-8, TNF-ɑ and IL-β. (DOCX)
