# Supplementary material for: The anti-apoptotic and anti-inflammatory effect of Lactobacillus acidophilus on Shigella sonnei and Vibrio cholerae interaction with intestinal epithelial cells: A comparison between invasive and non-invasive bacteria
Source: PLoS One. 2018 Jun 6;13(6):e0196941. doi: 10.1371/journal.pone.0196941 (PMC5991357; doi:10.1371/journal.pone.0196941)
Supplement: S1 Table — (DOCX) [file pone.0196941.s001.docx]

**S1 Table: Quantitative expression of IL-8, TNF-ɑ and IL-β.**

**A)**

**B)**

**C)**
